# Risk factors of malnutrition in Chinese children with congenital heart defect

**DOI:** 10.1186/s12887-020-02124-7

**Published:** 2020-05-13

**Authors:** Mingjie Zhang, Liping Wang, Rui Huang, Chongrui Sun, Nan Bao, Zhuoming Xu

**Affiliations:** 1 Cardiac Intensive Care Unit, Department of Thoracic and Cardiovascular Surgery, Shanghai Children’s Medical Center, Shanghai Jiao Tong University School of Medicine, 1678 Dongfang Road, Shanghai, 200127 China; 2 Department of Pediatric Surgery, Shanghai Children’s Medical Center, Shanghai Jiao Tong University School of Medicine, 1678 Dongfang Road, Shanghai, 200127 China

**Keywords:** Congenital heart disease, Cardiac surgery, Children, Malnutrition

## Abstract

**Background:**

The study aimed to investigate the risk factors of malnutrition in children with congenital heart defect (CHD) in China.

**Methods:**

This cohort study was performed at the biggest pediatric heart center in China; 3252 patients with CHD who underwent cardiac surgeries in 2013 were included. Anthropometric measurements included weight for age Z score (WAZ), weight for height Z score (WHZ), and height for age Z score (HAZ). The patients were classified as normal nutritional status and malnutrition, based on a cut-off Z score of <− 2. Factors associated with malnutrition were determined using logistic regression analysis.

**Results:**

The prevalence of preoperative WAZ < -2 (underweight), HAZ < -2 (stunting), and WHZ < -2 (wasting) was 23.3, 23.3, and 14.3%, respectively. The multivariable analysis of preoperative malnutrition showed that hospitalization, age at surgery, risk adjustment for congenital heart surgery-1 > 3, mechanical ventilation, pulmonary hypertension, and acyanotic heart disease were associated with underweight. Parents’ height, single ventricle, and cyanotic heart disease were associated with stunting. Hospitalization and pulmonary hypertension were associated with wasting. After surgery, the patients presented a significant improvement in growth within the first year in all three parameters and grew to the normal range of WAZ (− 0.3 ± 0.9, *P* < 0.001), HAZ (0.2 ± 0.8, *P* = 0.001), and WHZ (0.03 ± 0.6, *P* < 0.001) at 2 years after surgery. The prevalence of underweight, stunted, and wasting declined to 3.2, 2.7, and 1.9% 3 years after surgery. Malnutrition after surgery was associated with cardiac residual cardiac abnormalities (OR = 35.3, *p* < 0.0001), high Ross classification of heart function (OR = 27.1, *p* < 0.0001), and long-term taking oral diuretics (OR = 20.5, *P* = 0.001).

**Conclusions:**

Malnutrition is still a problem in children with CHD in China, especially before the surgery. There is need to strengthen the nutrition support for children with CHD before surgery. Hemodynamic factors were found to be the risk factors associated with malnutrition after operation.

## Background

Globally, congenital heart defect (CHD) represents one-third of all major congenital anomalies and are often associated with malnutrition and failure to thrive in children [1]. Growth failure in CHD has a multifactorial etiology, including genetic factors, chronic cyanosis, congestive heart failure, and pulmonary hypertension [2–5], as well as an inability to feed properly [6] and, therefore, inadequate nutrient intakes [7]. In fact, cardiac-related malnutrition is a major challenge affecting an estimated 50–90% of children with symptomatic CHD in developing countries [8]. Congestive heart failure causes increased energy requirements because of increased myocardial and respiratory work and poor feeding ability. Furthermore, growth failure in children with CHD has been associated with adverse developmental outcomes [9] and increased morbidity and mortality [10].

In developed countries, advancements in pediatric cardiac care, early prenatal and postnatal diagnosis, and timely corrective interventions for cardiac lesions have improved the outcomes of those children, but malnutrition still remains a challenge. In a recent paper from the UK, Marino et al. [11] reported that 28.2% of infants were stunted pre-operatively. In a study from Australia, 23% of children were underweight, and 21% were wasted [12]. In many countries, pediatric cardiac programs are not fully established, and follow-up data on CHD-related morbidity and mortality are lacking [13–15].

In China, as in many developing countries, the epidemiological data on CHD-related malnutrition are not clear. There might be many more risk factors associated with malnutrition than in developed countries. Therefore, this study aimed to investigate the effect and risk factors of malnutrition in children with CHD in China.

## Methods

### Patients

Children who underwent radical or palliative surgery for CHD at the Shanghai Children’s Medical Center (School of Medicine, Shanghai Jiaotong University) between January 2013 and December 2013 were retrospectively selected from the medical charts and prospectively followed up. The study was approved by the ethics committee of the Shanghai Children’s Medical Center.

The patients< 18 years for cardiac surgery were enrolled in. The exclusions criteria were: 1) gestational age < 36 weeks; or 2) a recognizable chromosomal or phenotypic syndrome associated with growth failure. 3252 children were enrolled in this study with median age of 275 days (range, 1–3671 days), median weight of 8 kg (range, 2–57 kg), and median height 70 cm.

### Data collection

Demographic and preoperative characteristics of the children, including sex, age, weight, height/length, diagnosis, preoperative percutaneous oxygen saturation (SpO_2_), and risk adjustment for congenital heart surgery-1 (RACHS-1) scores was collected. Pulmonary hypertension was diagnosed based on tricuspid valve regurgitation velocity of ≥3.5 m/s by echocardiography. The anthropometric measurements, including weight and height, were performed according to standard World Health Organization (WHO) procedures. Z-scores for weight for age (WAZ), weight for height (WHZ), and height for age (HAZ) were calculated using the anthropometric calculator module of WHO Anthro software (version 3.2.2, January 2011) based on the 2006 WHO child growth standards.

Genetic factors, including parents’ height, and socioeconomic factors such as family income, education levels of the parents, and residence were considered. The criterion for high family income was higher than the average of local income. The criterion for high education level of the parent was a bachelor or above.

The WHO global database on child growth and malnutrition recommends a cut-off Z-score < − 2 to classify low WAZ (underweight), low HAZ (stunting), and low WHZ (wasting) as malnutrition [9]. After the calculation of WAZ, WHZ, and HAZ, the patients were classified into normal nutritional status or malnutrition.

The patients with malnutrition were followed once a year for 3 years after the surgery, including growth, SpO_2_, residual cardiac abnormalities, Ross classification of heart function for children [10], and oral diuretics prescription. Cardiac residual abnormalities were diagnosed as residual shunt and obstruction of the tract or the vessels or moderate or severe regurgitation of the valve. Patients taking oral diuretics for more than 1 year were considered as long-term oral diuretics treatment.

### Statistical analysis

The data was analyzed using SPSS 20.0 (IBM, Armonk, NY, USA). Data with a normal distribution was presented as means ± standard deviations (SD). The mean of the two groups was compared by the t-test. Non-normally distributed values were presented as medians and ranges, and the medians of two groups were compared using the Mann-Whitney U test. Categorical data were represented as frequencies and percentages, and the chi-square test was used for testing. Odds ratios (ORs) with 95% confidence intervals (CIs) and *P*-values were computed by multivariate logistic regression analysis. A comparison of follow-up data with baseline values was made using the paired t-test. A P-value of < 0.05 was considered statistically significant.

## Results

### Characteristics of the patients

A total of 3279 patients were screened, and 3252 children meeting the inclusion criteria were included. The median age was 275 days (range, 1–3671 days); the median weight was 8 kg (range, 2–57 kg), and the median height was 70 cm (range, 40–146 cm). The top three CHD types were ventricular septal defect and atrial septal defect (49.6%), tetralogy of Fallot (11.6%), and pulmonary atresia (4.1%). 1863 (57.3%) children were male, and 1389 (42.7%) were female. There were more patients with cyanotic CHD (61.1% vs 30.1%, *p* < 0.0001) and single ventricle (11.8% vs 7.4%, *p* < 0.0001) in the female group. The HAZ (− 0.8 ± 1.7 vs − 1.6 ± 1.8, *p* < 0.0001) and WHZ (− 0.4 ± 1.6 vs − 0.6 ± 1.6, *p* = 0.001) were lower in the female group compared with the male group. The prevalence of underweight, stunting, and wasting was 23.3, 23.3, and 14.3%, respectively (Table 1). 60.9% of the malnutrition patients were male and 39.1% of them were female. Patients with age < 1 year were prone to malnutrition with high proportion of WAZ < -2(73.4%), HAZ < − 2(45%) and WHZ < − 2(41.2%) compared with other age subgroups (*P* < 0.0001). (Table 2).

**Table 1:** Clinical features and malnutrition status of children with CHD

Variable	Patients	Male	Female	*P*
Sex	3252	1863 (57.3%)	1389 (42.7%)	
Age(d)	275 (1–3671)	274 (1–3671)	289 (2–3584)	0.3
Weight (kg)	8 (2–57)	8 (2–56)	7.9 (2–57)	0.06
Height (cm)	70 (40–146)	70 (40–146)	70 (40–142)	0.56
Cyanotic CHD	1408 (43.3%)	560 (30.1%)	848 (61.1%)	< 0.0001
Acyanotic CHD	1844 (56.7%)	1303 (69.9%)	541 (38.9%)	
SV	303 (9.3%)	139 (7.4%)	164 (11.8%) 4	< 0.0001
Bi-V	2949 (90.7%)	1724 (92.6%)	1225 (88.2%)	
WAZ	−1.0 ± 1.5	−0.9 ± 1.5	−0.1 ± 1.5	0.48
WAZ < -2	758 (23.3%)	431 (23.1%)	327 (23.5%)	0.79
HAZ	−0.9 ± 1.8	−1.6 ± 1.8	−0.8 ± 1.7	< 0.0001
HAZ < -2	758 (23.3%)	426 (22.9%)	332 (23.9%)	0.49
WHZ	−0.5 ± 1.6	−0.6 ± 1.6	− 0.4 ± 1.6	0.001
WHZ < -2	466 (14.3%)	287 (15.4%)	179 (12.9%)	0.04
VSD and ASD	1614 (49.6%)	712 (38.2%)	902 (64.9%)	0.009
Tetralogy of Fallot	376 (11.6%)	175 (9.4%)	197 (14.2%)	
Pulmonary atresia	134 (4.1%)	77 (4.1%)	57 (4.1%)	

Data of age, weight, height was presented as median (min-max), data of WAZ, HAZ and WHZ was presented as mean ± standard deviations (x ± s), and other parameters in the table were presented as number (percentage)

*CHD* congenital heart defect, *SV* single ventricle, *Bi-V* biventricle, *WAZ* weight for age Z score, *HAZ* height for age Z score, *WHZ* weight for height Z score, *VSD* ventricular septal defect, *ASD* atrial septal defect

**Table 2:** Number (%) of patients with growth faltering in different age groups

	< 1 year	> = 1,< 3 years	> = 3, <=6 years	> 6 years	
WAZ < − 2	556 (73.4%)	143 (18.9%)	40 (5.3%)	19 (2.5%)	χ2 = 183.2
HAZ < − 2	341 (45%)	231 (30.5%)	122 (16.1%)	64 (8.4%)	P < 0.0001
WHZ < −2	192 (41.2%)	153 (32.8%)	71 (15.2%)	50 (10.7%)	

### Factors associated with pre-operative malnutrition

With regard to the pre-operative malnutrition, multivariable logistic regression analysis revealed several related risk factors. Hospitalization (OR = 4.1, 95%CI: 1.5–8.2, *P* = 0.03), mechanical ventilation (OR = 3.2, 95%CI: 2.5–8.9, *P* = 0.02), pulmonary hypertension (OR = 2.8, 95%CI: 1.6–5.1, *P* = 0.007), acyanotic heart disease (OR = 1.7, 95%CI: 0.9–3.0, *P* = 0.04), RACHS-1 > 3 (OR = 7.2, 95%CI: 1.3–27.0, *P* = 0.001), and age at surgery (OR = 0.7, 95%CI: 0.5–0.9, *P* = 0.02) were associated with underweight. RACHS-1 > 3 had the strongest association. Single ventricle (OR = 4.9, 95%CI: 1.5–17.0, *P* = 0.009), cyanotic heart disease (OR = 3.5, 95%CI: 1.4–6.5, *P* = 0.01), and parents’ height (OR = 9.5, 95%CI: 2.1–15.4, *P* = 0.005) were associated with stunting. Parents’ height had the strongest association. Hospitalization (OR = 2.9, 95%CI: 1.2–4.7, *P* = 0.03) and pulmonary hypertension (OR = 6, 95%CI: 0.5–9.9, *P* = 0.002) were associated with wasting. Pulmonary hypertension had the strongest association (Table 3).

**Table 3:** Multivariable logistic regression of predictors of pre-operative malnutrition

Factors		OR (95% CI)	*P*
WAZ < -2	Hospitalization	4.1 (1.5, 8.2)	0.03
	Age at surgery	0.7 (0.5, 0.9)	0.02
	RACHS-1 > 3	7.2 (1.3, 27.0)	0.001
	Mechanical ventilation	3.2 (2.5, 8.9)	0.02
	Pulmonary hypertension	2.8 (1.6, 5.1)	0.007
	Acyanotic heart disease	1.7 (1.9, 3.0)	0.04
HAZ < -2	Parents’ height	9.5 (2.1, 15.4)	0.005
	Single ventricle	4.9 (1.5, 17.0)	0.009
	Cyanotic heart disease	3.5 (1.4, 6.5)	0.01
WHZ < -2	Hospitalization	2.9 (1.2, 4.7)	0.03
	Pulmonary hypertension	6 (1.5, 9.9)	0.002

*OR* odds ratio, *CI* confidence interval, *WAZ* weight for age Z score, *RACHS-1* risk adjustment for congenital heart surgery-1, *HAZ* height for age Z score, *WHZ* weight for height Z score

### Post-operative malnutrition and growth trends

**Fig. 1:**
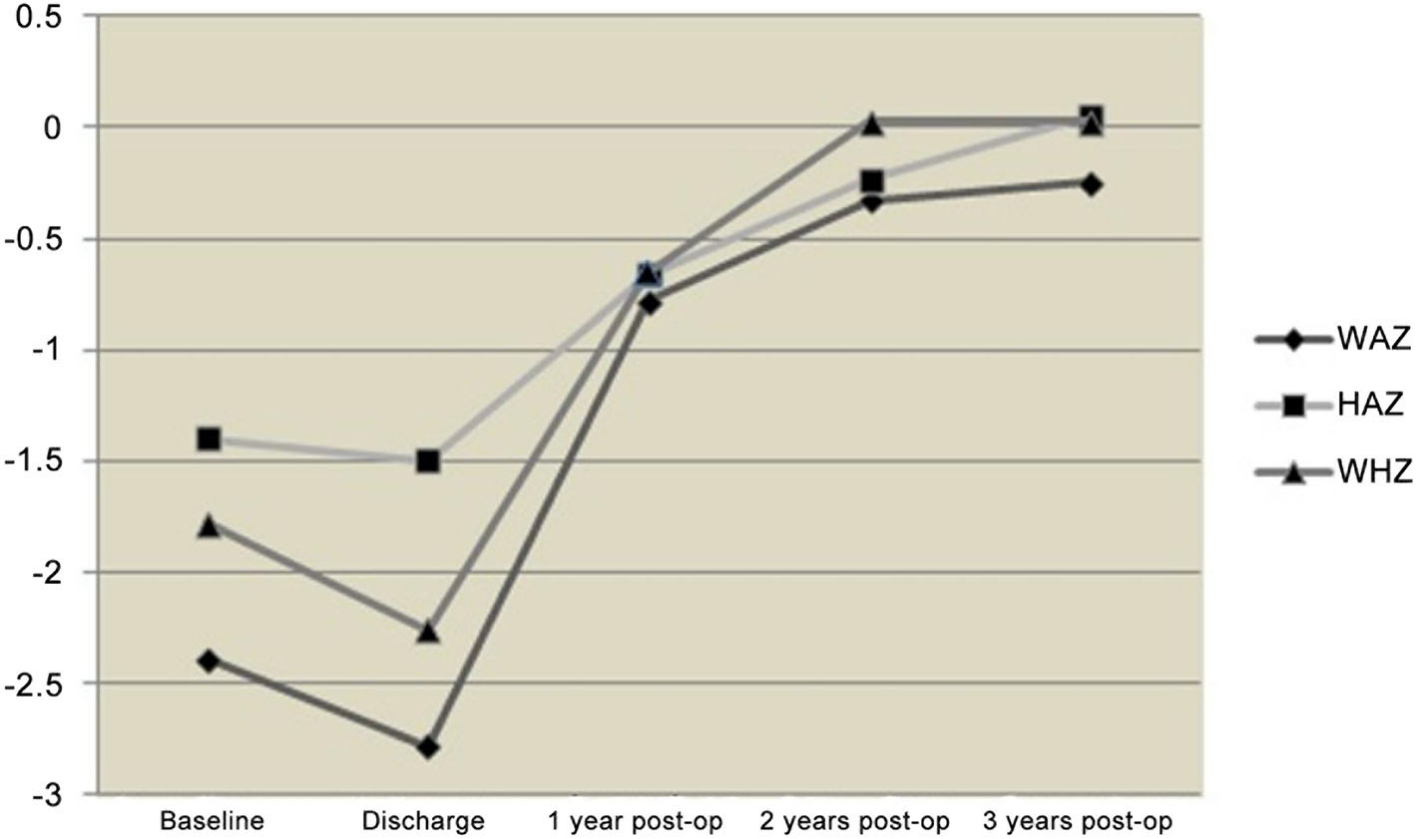
Trends in Z-scores before and after operation. Decline was seen in WAZ (− 2.7 ± 1.2, *P* = 0.04) at discharge. The most significant increase in all the three parameters of WAZ (− 0.8 ± 1.1, *P* = 0.04), HAZ (− 0.7 ± 1.3, *P* = 0.004), and WHZ (− 0.7 ± 1.3, *P* < 0.001) were observed at the end of the first year after surgery. The children presented a modest improvement in growth at the end of the second year, and the individual values could be considered within the normal range [(− 0.3 ± 0.9, *P* < 0.001), HAZ (− 0.2 ± 0.8, *P* = 0.001), and WHZ (0.03 ± 0.6, *P* < 0.001)]. There were no significant differences in the growth assessment data between the second year and third years

There were 107 in-hospital deaths among the 3252 (mortality rate of 3.3%). Among 3165 cases who were discharged, there were 790 malnutrition cases with WAZ < -2 or HAZ < -2, or WHZ < -2 followed after surgery. The 3-year follow-up data of 699 (88.5%) malnutrition patients were analyzed. There were 13 deaths (1.9%, nine after re-admission, and four at home). The growth trends of the survivors are presented in Fig. 1. It seemed that all the growth assessment data declined when the patients were discharged, but actually, the only significant decline was seen in WAZ (− 2.7 ± 1.2, *P* = 0.04). The most significant increase in all three parameters of WAZ (− 0.8 ± 1.1, P = 0.04), HAZ (− 0.7 ± 1.3, *P* = 0.004), and WHZ (− 0.7 ± 1.3, *P* < 0.001) were observed at 1 year after surgery. During the second year, the children also presented a modest improvement in growth, and the individual values could be considered within the normal range [WAZ (− 0.3 ± 0.9, P < 0.001), HAZ (− 0.2 ± 0.8, *P* = 0.001) and WHZ (0.03 ± 0.6, *P* < 0.001)]. There were no significant differences between years 2 and 3.

### Factors associated with postoperative malnutrition

There were still some underweight (22 cases, 3.2%), stunting (19 cases, 2.7%), and wasting (13 cases, 1.9%) children at the end of follow-up. The frequencies of palliative suegery, re-operation, residual anatomy, long-term oral diuretics, and Ross classification of 2–4 were significantly higher in the malnutrition group than in the normal nutrition group. More children lived in the suburban area in the malnutrition group, and their parent’s education was poor (Table 4). Three factors were associated with malnutrition after surgery: cardiac residual abnormalities (OR = 35.3, 95%CI: 5.7–215, *P* < 0.001), Ross classification of 3 and 4 (3: OR = 27.1, 95%CI: 4.7–155.6, *P* < 0.001; 4: OR = 20.5, 95%CI: 3.2–132.0, *P* = 0.001), and long-term oral diuretics treatment (OR = 5.179, 95%CI: 1.0–26.2, *P* = 0.047) (Table 5).

**Table 4:** Comparison the factors related to post-operative malnutrition

Factors	Normal nutrition (*n* = 677)	Malnutrition (*n* = 22)	*P*
Age (years), median (range)	6 (3–9)	5 (3–8)	0.63
Male, n (%)	384 (56.7)	13 (59.1)	0.82
Pre-op WAZ	−2.5 ± 0.8	−2.2 ± 0.9	0.13
Pre-op HAZ	−2.6 ± 1.0	−2.2 ± 0.7	0.09
Pre-op WHZ	−2.6 ± 1.1	−2.6 ± 1.2	0.83
Anatomy of the ventricles, *n* (%)			0.373
Single ventricle	56 (8.7)	3 (13.6)	
Double ventricles	621 (91.3)	19 (86.4)	
Surgery, *n* (%)			0.001
Radical	647 (95.6)	20 (90.9)	
Palliative	30 (4.4)	2 (9.1)	
Re-operation, *n* (%)	36 (5.3)	5 (22.7)	< 0.001
Residue anatomy, *n* (%)	7 (1.0)	11 (50)	< 0.001
High family income^a^, *n* (%)	77 (11.4)	4 (18.1)	0.326
High education level of parents^b^, *n* (%)	90 (13.2)	2 (9.1)	< 0.001
Residence, *n* (%)			< 0.001
Suburb	34 (5.0)	5 (22.7)	
Urban	643 (95.0)	17 (77.3)	
Parents’ heigt (cm)	167 ± 3.7	166 ± 3.2	0.09
Long-term oral diuretics^c^, *n* (%)	21 (3.1)	11 (50)	0.04
Ross classification of heart function, n (%)			< 0.001
Ross-1	568 (83.9)	4 (18.2)	
Ross-2	68 (10.0)	8 (36.4)	
Ross-3	17 (2.5)	5 (22.7)	
Ross-4	24 (3.5)	5 (22.7)	
Cyanotic heart disease, *n* (%)	34 (5.0)	2 (9.1)	0.395

*WAZ*, weight for age Z score, *HAZ* height for age Z score, *WHZ* weight for height Z score

a. High family income was defined as the income higher than the average of local income

b. High education level of parents was defined as a bachelor or above in one of the parents

c. Long-term oral diuretics was defined as more than 1 year of oral diuretics treatment

**Table 5:** Multivariable logistic regression of predictors for post-operative malnutrition

Factors	OR (95% CI)	*P*
Single or double ventricles		0.38
Radical or palliative surgery		0.20
Re-operation		0.52
Residue anatomy	35.3 (5.7, 215)	< 0.001
Parents’ education		0.90
Rural or urban residence		0.14
Long-term oral diuretics	5.179 (1.0, 26.2)	0.047
Ross classification of heart function		
Ross-1	1	
Ross-2	15.2 (3.3, 70.4)	0.94
Ross-3	27.1 (4.7, 155.6)	< 0.001
Ross-4	20.5 (3.2, 132.0)	0.001

## Discussion

The purpose of the study was to extend the epidemiological data about malnutrition and children’s growth in CHD to the Chinese context and explore the risk factors associated with malnutrition. Our study showed that 23.3% of children were underweight (WAZ < -2), 23.3% were stunted (HAZ < -2), and 14.3% were wasted (WHZ < -2) before the surgery, which was comparatively lower than that of other developing countries like Egypt, Uganda, and India [14–16]. Previous reports showed that CHD-related growth faltering malnutrition is particularly common in developing countries. For instance, in Egypt, Hassan et al. [14] reported the overall prevalence of malnutrition was 84.0% in patients with CHD pre-operatively. In South India, Vaidyanathan et al. [15] reported a high prevalence of underweight (59.0%) in children with CHD pre-operatively. In Uganda, a study showed that 31.5% of the children with CHD were wasted, 42.5% were underweight, and 45.4% were stunted [16]. On the other hand, in developed countries like France, Blasquez et al. [17] reported a quite low prevalence of malnutrition (15%) in CHD children, even in children younger than 6 months. The difference may be caused by the absence of efficient dietetic intervention before operation, and some of the children could not receive surgery immediately [18]. Studies from the United Kingdom and Australia reported similar incidence as in the present study, especially in Australia, where similar proportions of underweight (23%), stunted (21%), and wasting (18%) were reported [11, 12].

As shown in the present study, hospitalization, mechanical ventilation, pulmonary hypertension, acyanotic heart disease, RACHS-1 > 3 and age at surgery were associated with underweight. RACHS-1 > 3 had the strongest association, demonstrating that the severity of heart defects (including aortic arch plasty, arterial switch, pulmonary atresia, and other complex CHD) affects the degree of nutrition [18]. Interestingly, we found that older age at surgery might be a protective factor of malnutrition. As a matter of fact, with the development of echocardiography and surgical techniques in the local hospital, the patients admitted to our cardiac center are more severe cases and younger. Usually, the younger the patients were, the more serious the disease was. Our study also showed that patients under 1 year had a higher incidence of malnutrition with WAZ < -2 (73.4%), HAZ < -2 (45%) and WHZ < − 2 (41.2%), which indicated the infants were prone to malnutrition. Similarly, a previous study showed on the the predictors of malnutrition was age under 5 years [19] .

Single ventricle, cyanotic heart disease, and parents’ height were associated with being stunted, and parents’ height had the strongest impact. Hospitalization and pulmonary hypertension were associated with wasting, and pulmonary hypertension had the strongest association. Malnutrition is commonly seen in children with cyanotic disease, single ventricle, and pulmonary hypertension. In the present study, children with acyanotic CHD were more likely to be underweight, while those with cyanotic CHD were more likely to be stunted. Similarly, Radman et al. [5] also reported a decrease in weight gain velocity in acyanotic children, but Okoromah et al. [19] reported that children with acyanotic CHD were more likely to be wasted. Nevertheless, our findings on cyanotic CHD and its association with being stunted has previously been reported in the literature. Linde et al. [20] reported that both wasting and stunting were more common in cyanotic CHD than that in acyanotic CHD.

After surgery, malnutrition patients showed dramatic decreases in all parameters. The patients need more energy in the early period after surgery, but their gut function is not good enough to get the energy [21]. In addition, fluid intake is restricted strictly to avoid volume overload in order to protect heart function, and diuretics are usually given [21]. Another interesting finding is that the maximum catch-up growth occurs in the first year. Study from developed countries showed that catch up growth is largely complete within 2 years after surgery [22]. This may suggest that growth is less influenced by the cardiac condition itself after a certain period after correction and that environmental, dietary, and genetic factors may be more important once the cardiac condition is corrected. Nevertheless, there were still some children malnourished at the end of the third year after surgery (3.2% underweight, 2.7% stunting, and 1.9% wasting). In contrast with the present study, Vaidyanathan et al. from India [15] reported that malnutrition persisted in 27.3% of patients on follow-up 2 years after surgery, which is higher than in the present study but their incidence of pre-operative malnutrition (59.0%) was also higher.

Only three factors were associated with malnutrition by multivariable logistic regression analysis: residual abnormalities, Ross classification of 3 and 4, and long-term oral diuretics treatment. Those results suggest that correction of the anatomy is the key point for the somatic development of the children.

This study has some limitations. It was carried out at a highly developed and specialized center with substantial expertise and, therefore, might not reflect the entire situation in China. Some studies in other provinces showed the incidence of malnutrition was in the range of 25.3–47.1% [23, 24], which indicated the problem of malnutrition might be more serious in the undeveloped area. Besides there is no follow-up data about neural development, which might have a close relationship with malnutrition [25]. The nutrition supplement and daily diet after surgery were not collected, which might introduce the incomprehensive analysis of the associated factors with post-surgery malnutrition. Future studies should explore the long-term impacts of improved nutrition on neural development before and after corrective interventions for CHD in China.

## Conclusions

Malnutrition is a very common complication in children with symptomatic CHD. About 25% of the children with CHD suffered from malnutrition. Hospitalization, mechanical ventilation, pulmonary hypertension, acyanotic heart disease, RACHS-1 > 3, and age at surgery were associated with preoperative malnutrition. Most of the survivors would have normal development after anatomical correction, but some patients were still malnourished 3 years after surgery. Incomplete anatomical correction could be associated with malnutrition after surgery.

## Data Availability

Data can be made available on request and following institutional and ethic board approvals for release.

## Abbreviations

CHD: Congenital heart defect
WAZ: Weight for age Z score
WHZ: Weight for height Z score
HAZ: Height for age Z score
VSD: Ventricular septal defect;
ASD: Atrial septal defect
RACHS-1: Risk adjustment for congenital heart surgery-1
